# The role of loneliness in perceived mastery and life satisfaction among older adults

**DOI:** 10.1093/geroni/igaf122.3125

**Published:** 2025-12-31

**Authors:** Inhye Jung, Hyo Jung Lee

**Affiliations:** Yonsei University, Seoul, Republic of Korea; Yonsei University, Seoul, Republic of Korea

## Abstract

This study examines the impact of loneliness on older adults’ quality of life from a relational autonomy perspective. From this perspective, an individual’s perceived social connectedness can influence their psychological and behavioral health. Considering loneliness as a negative aspect of perceived social connectedness, this study explores its impact on perceived autonomy and health status. Utilizing data from the 2018 Health and Retirement Study (HRS) (N = 2,279), we conducted multiple mediation analyses using PROCESS Macro Model 6 to assess whether these factors explain the association between loneliness and subjective well-being. The results indicate that higher levels of loneliness are significantly associated with lower perceived mastery (b = -0.7107, p < .001), which, in turn, is related to poorer self-rated health and lower life satisfaction. Overall, the total indirect effect of loneliness on life satisfaction is significant (b = -0.2286, 95% CI [-0.2918, -0.1741]). Among the mediation pathways, the primary pathway (Loneliness → Perceived Mastery → Life Satisfaction) shows a significant indirect effect (b = -0.19, 95% CI [-0.2520, -0.1388]). Additionally, a smaller but significant mediation pathway (Loneliness → Perceived Mastery → Self-Rated Health → Life Satisfaction) further contributes to the overall effect (b = -0.0131, 95% CI [-0.0208, -0.0071]). These findings underscore the detrimental effects of loneliness on older adults’ psychological and physical well-being, highlighting the need for interventions that enhance an individual’s sense of control over their lives and foster a more informed perception of their well-being.

